# NIS expression in thyroid tumors, relation with prognosis clinicopathological and molecular features

**DOI:** 10.1530/EC-17-0302

**Published:** 2017-11-09

**Authors:** Catarina Tavares, Maria João Coelho, Catarina Eloy, Miguel Melo, Adriana Gaspar da Rocha, Ana Pestana, Rui Batista, Luciana Bueno Ferreira, Elisabete Rios, Samia Selmi-Ruby, Bruno Cavadas, Luísa Pereira, Manuel Sobrinho Simões, Paula Soares

**Affiliations:** 1 Instituto de Investigação e Inovação em Saúde (i3S)Porto, Portugal; 2 Institute of Molecular Pathology and Immunology of the University of Porto (IPATIMUP)Porto, Portugal; 3 Medical Faculty of the University of PortoPorto, Portugal; 4 Institute of Biomedical Sciences of Abel Salazar (ICBAS)Porto, Portugal; 5 Department of EndocrinologyDiabetes and Metabolism, University and Hospital Center of Coimbra, Coimbra, Portugal; 6 Medical FacultyUniversity of Coimbra, Coimbra, Portugal; 7 Public Health UnitACeS Baixo Mondego, Coimbra, Portugal; 8 Department of PathologyMedical Faculty of the University of Porto, Porto, Portugal; 9 Department of PathologyHospital de S. João, Porto, Portugal; 10 Inserm UMR-S1052CNRS UMR5286, Centre de Recherche en Cancérologie de Lyon, Lyon, France

**Keywords:** thyroid, cancer, NIS, *SLC5A5*, immunohistochemistry

## Abstract

Thyroid cancer therapy is based on surgery followed by radioiodine treatment. The incorporation of radioiodine by cancer cells is mediated by sodium iodide symporter (NIS) (codified by the *SLC5A5* gene), that is functional only when targeted to the cell membrane. We aimed to evaluate if NIS expression in thyroid primary tumors would be helpful in predicting tumor behavior, response to therapy and prognosis. NIS expression was addressed by qPCR and immunohistochemistry. In order to validate our data, we also studied *SLC5A5* expression on 378 primary papillary thyroid carcinomas from The Cancer Genome Atlas (TCGA) database. In our series, *SLC5A5* expression was lower in carcinomas with vascular invasion and with extrathyroidal extension and in those harboring *BRAF*V600E mutation. Analysis of *SLC5A5* expression from TCGA database confirmed our results. Furthermore, it showed that larger tumors, with locoregional recurrences and/or distant metastases or harboring *RAS*, *BRAF* and/or *TERT* promoter (*TERT*p) mutations presented significantly less *SLC5A5* expression. Regarding immunohistochemistry, 12/211 of the cases demonstrated NIS in the membrane of tumor cells, those cases showed variable outcomes concerning therapy success, prognosis and all but one were wild type for *BRAF*, *NRAS* and *TERT*p mutations. *SLC5A5* mRNA lower expression is associated with features of aggressiveness and with key genetic alterations involving *BRAF*, *RAS* and *TERT*p. Mutations in these genes seem to decrease protein expression and its targeting to the cell membrane. *SLC5A5* mRNA expression is more informative than NIS immunohistochemical expression regarding tumor aggressiveness and prognostic features.

## Introduction

Sodium iodide symporter is a transmembrane glycoprotein (codified by the *SLC5A5* gene) expressed almost exclusively in the basolateral plasma membrane of thyroid follicular cells. It plays a central role in thyroid metabolism, mediating the active transport of iodine from the bloodstream into the follicular cells, the first step for thyroid hormones’ synthesis. NIS plays an essential role in the treatment of differentiated thyroid carcinomas (DTC), which usually maintain NIS expression, allowing the recognition and the treatment of recurrences and metastases with radioactive iodine (RAI) (1). Nonetheless, a significant subgroup of DTC patients with advanced disease lose NIS expression and become refractory to ^131^I; some of these patients die within 3–5 years (2). Furthermore, a study performed by Yildririm-Poyraz and coworkers (3) demonstrated that NIS expression in nontumoral thyroid tissues associates with higher rates of delayed structural response. NIS expression has been widely studied in normal thyroid and tumor tissues, on one hand to verify if its downregulation could be the molecular cause for the decrease of RAI uptake and on the other hand to understand the impairing mechanisms of NIS expression and function. However, no clear answer emerged from the results obtained in the previous studies. Despite the central role of NIS in diagnosis, treatment and follow-up of thyroid cancer patients, reliable methods for ascertaining NIS expression and functionality in clinical samples are not available.

In the majority of the studies, *SLC5A5* mRNA levels are lower in thyroid carcinomas than in adenomas (4) and normal adjacent thyroid (5, 6, 7); furthermore, *SLC5A5* expression presents some limitations in predicting NIS expression and functionality: whereas a negative or low mRNA level may lead to reduced protein expression, a positive or high mRNA expression does not always correspond to higher protein levels or higher functionality (7, 8).

These observations suggest that in thyroid carcinomas, besides transcription regulation, NIS expression appears to be modulated by post-transcriptional events. Therefore, studies of NIS expression by immunohistochemistry (IHC) (1, 9, 10, 11, 12, 13, 14, 15, 16, 17, 18, 19, 20, 21, 22, 23, 24, 25), may be, theoretically, more informative since they ‘grab’ NIS a step forward in its biological processing and allow the evaluation of the localization of NIS in the basolateral plasma membrane of follicular cells (the functional transporter).

According to the published data, NIS expression (evaluated by IHC) varies in different thyroid tissues. In normal thyroid, it is low and very heterogeneous; only a few follicular cells within some follicles express NIS in the basolateral plasma membrane (10, 14, 17, 21, 26), suggesting that, NIS expression is tightly regulated in thyroid gland. In carcinomas, when NIS is present, it is usually expressed in a higher number of cells than in normal tissue and the expression is mainly intracytoplasmic, poorly targeted to the basolateral plasma membrane (1, 11, 12, 13, 14, 17, 21, 22, 23). The increased intracytoplasmic NIS staining in thyroid tumors compared to normal tissue has been pointed out as a reason for the decreased RAI uptake in tumors, reflecting a mislocalization of NIS from the basolateral membrane, which would impair its activity (17). This assumption has been questioned, because the real significance of intracytoplasmic NIS detected by immunostaining remains unclarified (21).

The molecular mechanisms responsible for the downregulation and/or not targeting to the basolateral membrane of NIS in thyroid tumors remain poorly understood, but some studies demonstrated that both mRNA and protein are differentially expressed according to the genetic background of the tumor. In fact, papillary thyroid carcinomas (PTCs) harboring the *BRAF*V600E mutation present lower *SLC5A5* mRNA and NIS protein expression as well as less targeting to the basolateral membrane compared to PTCs *BRAF*WT (19, 24, 27). Nonetheless, a recent study reported different association between NIS membrane expression and *BRAF*V600E mutation in a series of 96 cPTCs, demonstrating that the ones harboring *BRAF*V600E mutation expressed more often NIS in the cell membrane of tumor cells compared to *BRAF*WT cPTCs (28). Less is known about the impact of mutations in other genes (i.e. *RAS* and *TERT*p) on *SLC5A5* and NIS expression/targeting to the basolateral membrane.

NIS being the central molecule for DTC treatment, it is logical to study if its expression in the primary tumor would be helpful in predicting therapy response as well as tumor behavior and prognosis. Some studies tried to understand if NIS immunohistochemical expression in thyroid primary tumors would be helpful in predicting ^131^I uptake in recurrences and distant metastases. Although positive NIS immunostaining in primary tumors seemed to be predictive of positive recurrences and metastases on ^131^I scans, other studies did not distinguish whether NIS was expressed in the cell basolateral membrane, and negative NIS staining did not predict ^131^I scan-negative metastases (13, 15, 18). Even when only NIS membrane staining was considered, a negative NIS staining in the primary tumor was still not predictive of a negative ^131^scan of subsequent recurrences (29). To the best of our knowledge, there is only one study that addressed possible associations between NIS expression, evaluated by immunohistochemistry (IHC), and clinicopathological features and prognosis in a large series of thyroid primary tumors (1), reporting a significantly lower NIS expression in older patients (≥45 years) and also that NIS expression in the primary tumor was not useful as a prognostic marker.

So, in our opinion, more retrospective studies in larger series of primary tumors are still necessary, to understand the role of NIS expression in therapy response, tumor behavior and prognosis, and also if other factors besides *BRAF*V600E mutation can contribute to NIS downregulation and/or misdirecting to the basolateral membrane. Furthermore, it is also important to understand the advantages and limitations of the analysis of *SLC5A5* and NIS expression and evaluate what is the better/more informative method to study NIS expression.

Having this in mind, we addressed *SLC5A5* expression by qPCR and NIS expression by IHC analysis, in a large series of primary thyroid carcinomas and looked for possible associations with some clinicopathological and molecular features, as well as to the response to RAI therapy and outcome. In order to validate our results of *SLC5A5* mRNA expression associations’ with clinicopathological and molecular features and also to get new evidences, we used the data available about *SLC5A5* in TCGA Research Network that completed an integrated genomic analysis of 496 PTCs using NGS and other pan-genomic technologies, together with detailed pathologic and clinical data (30).

## Materials and methods

### Patient samples

Our series was composed by 255 thyroid samples from 229 patients. Cases were collected from the files of the Institute of Molecular Pathology and Immunology of the University of Porto (IPATIMUP, Porto, Portugal), corresponding to patients with thyroid tumors (*n* = 229) operated and followed in two university hospitals. Samples from normal thyroid (*n* = 25) and Graves’ disease (*n* = 1) were obtained from the contralateral lobe of the surgical specimens. Carcinomas series was composed by 193 PTCs (123 cases of classical PTC (cPTC), 47 cases of follicular variant of PTC (fvPTC) and 23 cases of other PTC variants), 23 follicular thyroid carcinomas (FTC) and 13 poorly differentiated thyroid carcinomas (PDTC). In 166 of the cases, there was only formalin-fixed paraffin-embedded (FFPE) representative tissue; in 45 cases, there were FFPE samples and correspondent frozen tissue (the tumors were divided at the time of surgery) and in 18 cases, there was only frozen tissue available. Frozen material was collected at the time of surgery and conserved at −80°C. The histology of all tumor samples was reviewed by three pathologists (CE, ER, MSS) according to the criteria of the World Health Organization (31). Clinicopathological and molecular data of the 229 patients with carcinoma are summarized in Supplementary Table 1 (see section on supplementary data given at the end of this article). In 141 cases, follow-up data were available. The number of ^131^I treatments varied from 1 to 5 treatments (mean 1.9), and the cumulative total dose of RAI was between 30 and 1146 mCi (mean 251 mCi). This work was approved by the Ethic Committee for Health (CES) of the Hospital Center of São João (CHSJ)/Faculty of Medicine of the University of Porto (FMUP) (CES 137 284–13) and by the Ethic Committee of the Faculty of Medicine of the University of Coimbra (n° 1309). All the procedures described in this study were in accordance with national ethical standards (Law n° 12/2005) and Helsinki declaration. Patients signed an informed consent form.

### Patient follow-up

Patients were treated and followed in accordance with the international protocols available at the time. Data regarding the number of radioiodine treatments and cumulative activity were retrieved from hospital records. Patients were considered as being disease-free at the end of follow-up if they had undetectable stimulated thyroglobulin (in the absence of thyroglobulin antibodies), and no evidence of the disease on radiographic or radionuclide imaging. Follow-up time (in years) varied from 0.3 up to 38.9 years, with a mean value of 8.0 ± 6.6 years. For statistical analysis, we defined the category ‘additional treatments’, in which we included other treatment modalities in addition to radioiodine, including extra surgery, external beam irradiation and treatment with tyrosine kinase inhibitors.

### Dataset PTC in TCGA

There were 378 tumor cases for which there was information for the main driver somatic mutations (*TERT*p, *BRAF* and *RAS*), gender and *SLC5A5* expression. Of these, we eliminated 4 cases, for which the *SLC5A5* expression was above the 99 percentile, being outliers. A total of 353 of the cases had information about tumor size, 362 had information for extrathyroidal extension, 282 had information for lymph node metastases (at the time of diagnosis) and all 374 had information about new tumor event (lymph node metastases or local recurrence (grouped in locoregional recurrence) and distant metastases). The *SLC5A5* expression was inferred from RNA-seq data and quantification reflects reads per kilobase per million mapped reads (RPKM).

There were also 58 *SLC5A5* expression measures in adjacent tissue of the PTC cases, and two of them were not considered for further analyses as values were above the 99 percentile.

### DNA extraction, PCR and Sanger sequencing

DNA extraction from FFPE tissues was performed from 10 μm sections after careful microdissection. DNA extraction was performed using Ultraprep tissue DNA kit (AHN Biotechnologie, Nordhausen, Germany) following the manufacturer’s instructions. The genetic characterization of part of the tumors regarding *BRAF*, *NRAS* and *TERT* promoter mutations (*TERT*p) had been reported previously; mutations were screened as previously described (32, 33, 34).

### RNA extraction and reverse transcription

Total RNA was extracted from tumors and from contralateral normal adjacent thyroid, from which frozen samples were available (*n* = 84), using a TRIzol commercial kit (Thermo Scientific/GIBCO) according to the manufacturer’s protocol. RNA was quantified by spectrophotometry, and its quality was checked by analysis of 260/280 nm and 260/230 nm ratios. For cDNA preparation, 1 μg of total RNA was reverse-transcribed using the RevertAid first-strand cDNA synthesis kit (Thermo Scientific/Fermentas).

### Real-time PCR

Reverse transcription products were amplified for the *SLC5A5* gene and detected by a probe (IDT: Integrated DNA Technologies, Leuven, Belgium; no. HS.PT.56a.40789288), as previously described (35).

### Immunohistochemistry

Immunohistochemistry was performed in normal thyroid and in 211 carcinomas. Briefly, deparaffinized and rehydrated sections were subjected to heat-induced antigen retrieval in 10 mM sodium citrate buffer (pH 6.0). Endogenous peroxidase activity was blocked with 3% of hydrogen peroxide and nonspecific binding with Large Volume Ultra V Block reagent (Thermo Scientific/Lab Vision). Sections were then incubated overnight at 4°C with anti-NIS antibody (1:400) clone FP5A (Thermo Scientific/Lab Vision) and in 24 carcinomas with anti-NIS pAb 795 IgG (20 µg/mL) (kindly supplied by Dr Ruby) (36). Additionally, Tyramide Signal Amplification (TSA) Biotin System (Perkin-Elmer) was used for signal amplification in 44 carcinomas, according to manufacturer’s instructions. The detection was performed with a labeled, streptavidin–biotin immunoperoxidase detection system (Thermo Scientific/Lab Vision) followed by 3,3′-diaminobenzidine (Dako) and counterstained with hematoxylin. Graves’ disease sample was used as a positive control and the negative control consisted in omission of the primary antibody.

Slides were evaluated by two observers and were analyzed according to the percentage of tumor-stained cells, the intensity and the cellular localization of the staining. In order to compare our results to the literature, we considered cases with >5% of stained tumor cells (regardless of the cellular localization) as positive. Nevertheless, all our statistical analyses were performed considering two groups; cases that presented membrane staining in tumor cells and all the other cases. Photographs were acquired using Nikon DS-L1 camera in 100× and 400× magnifications.

### Statistical analysis

Statistical analysis was performed using 21.0 SPSS Statistical Package (SPSS, 2003). Fisher’s exact test and independent-samples *t*-test were performed to correlate NIS and *SLC5A5* mRNA expression with clinicopathological and molecular features. When parametric tests were not applicable, we used alternative tests, specifically Mann–Whitney (independent samples). Wilcoxon (related samples) was used to compare *SLC5A5* expression between tumor samples and their adjacent normal counterparts. Kruskal–Wallis test was used to correlate *SLC5A5* expression (retrieved from TCGA and database) with clinicopathological and molecular features. Values of *P* ˂ 0.05 were considered statistically significant.

## Results

### *SLC5A5* mRNA expression

**Figure 1:**
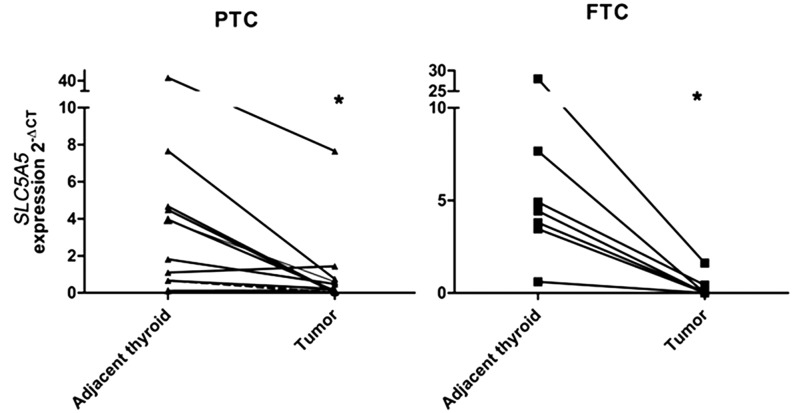
*SLC5A5* mRNA expression in thyroid carcinomas and paired normal adjacent counterparts.

*SLC5A5* expression was significantly lower in carcinomas than that in normal adjacent counterparts (Fig. 1). No significant difference was observed between the three different carcinoma histotypes (PTC, FTC and PDTC). Considering the analysis in DTC, *SLC5A5* expression was significantly lower in males and in cases with vascular invasion (*P* = 0.003 and *P* = 0.03, respectively) (Table 1). *SLC5A5* expression in normal thyroid from males was not significantly different from that of females (data not shown). In addition, there was a tendency to lower *SLC5A5* levels in cases with extrathyroidal extension (*P* = 0.06) and in PTCs harboring *BRAF*V600E mutation (*P* = 0.07). When the statistical analysis was performed only in the PTC group, all the significant associations described in the DTC group were maintained.

**Table 1:** Associations between *SLC5A5* mRNA expression with clinicopathological and molecular features in DTCs.

		***SLC5A5* expression**	***P* value**
Gender	F (*n* = 47)	1.2 ± 2.2	
	M (*n* = 12)	0.2 ± 0.2	0.003
Age	<45 years (*n* = 30)	1.0 ± 1.5	
	≥45 years (*n* = 29)	1.1 ± 2.4	0.8
Tumor capsule	Present (*n* = 27)	1.1 ± 1.6	
	Absent (*n* = 30)	0.7 ± 1.6	0.4
Tumor capsule invasion	Yes (*n* = 17)	0.9 ± 1.6	
	No (*n* = 11)	1.4 ± 1.4	0.4
Extrathyroidal extension	Yes (*n* = 17)	0.5 ± 1.1	
	No (*n* = 37)	1.4 ± 2.4	0.06
Lymphocytic infiltration	Present (*n* = 19)	0.9 ± 1.9	
	Absent (*n* = 37)	1.2 ± 2.2	0.7
Vascular invasion	Present (*n* = 28)	0.4 ± 0.8	
	Absent (*n* = 29)	1.5 ± 2.6	0.03
Lymph node metastases	Present (*n* = 13)	0.5 ± 0.8	
	Absent (*n* = 18)	0.4 ± 0.7	0.8
BRAF*	WT *n* = (27)	1.6 ± 2.7	
	V600E (*n* = 20)	0.5 ± 1.0	0.07
*NRAS*	WT (*n* = 54)	1.0 ± 2.0	
	Mut (*n* = 6)	1.3 ± 1.7	0.7

*PTC only.

### *SLC5A5* mRNA expression (TCGA database)

**Figure 2:**
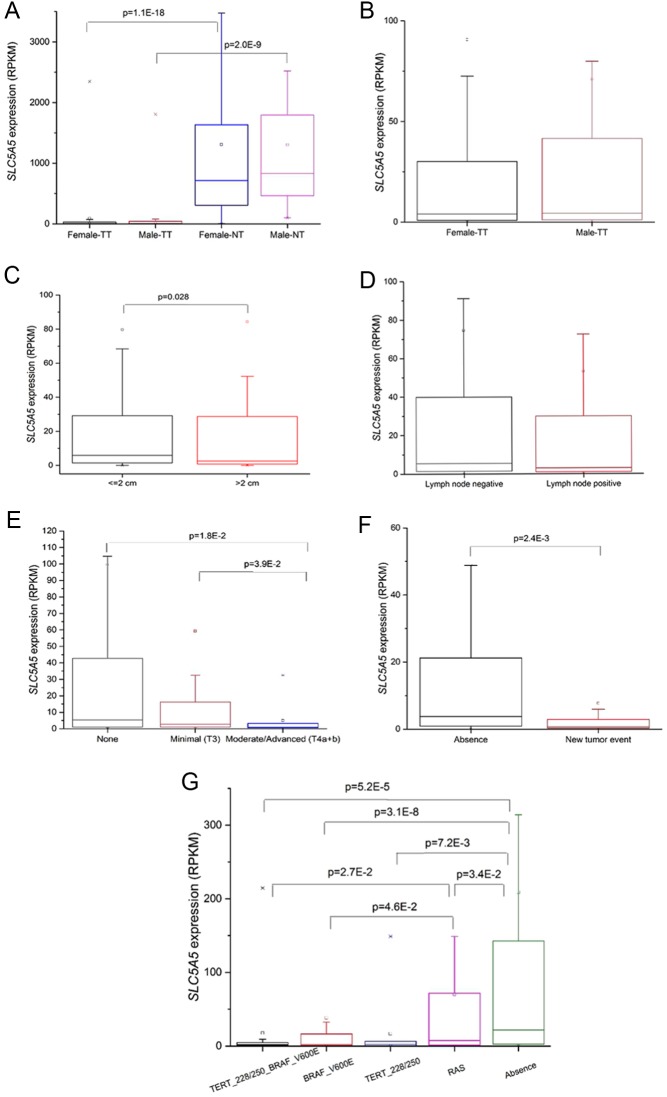
*SLC5A5* expression in primary PTCs (RPKM), data retrieved from TCGA database. Comparative analysis of *SLC5A5* expression. (A) Between genders in tumor (TT) and normal tissue (NT); (B) between genders only in tumor tissue (TT); (C) in tumors with ≤2 cm and >2 cm; (D) in cases with or without lymph node metastases at the time of diagnosis; (E) in cases without, with minimal (T3) and with moderate/advanced extrathyroidal extension (T4a+b); (F) in cases with and without recurrence and (G) between cases with different genetic backgrounds (WT, *RAS* mutation, *TERT*p mutation, *BRAF* mutation, *BRAF* + *TERT*p mutation). The boxes represent the interquartile range; the whiskers are the 5% and 95% quartiles; the small open boxes are the mean values and the lines are the median values. Significant values for the Kruskal–Wallis test are indicated.

The *SLC5A5* expression was around 200 times higher in normal tissue than in tumor tissue in both genders, but no differences in tumor and in adjacent tissue between genders were found (Fig. 2A and  B). *SLC5A5* expression was significantly higher in smaller tumors ≤2 cm (median = 5.85) compared to those with >2 cm (median = 2.51) (*P* = 0.028; Fig. 2C). There was no statistical difference in *SLC5A5* expression in primary tumors with (median = 3.0) or without (median = 5.4) lymph node metastases at the time of diagnosis (*P* = 0.253) (Fig. 2D).

The *SLC5A5* expression was reduced with the level of the extrathyroidal extension (median values: 5.4 for ‘none’; 2.8 for ‘minimal (T3)’ and 0.9 for ‘moderate/advanced (T4a + b)’), reaching statistical significance for comparisons between ‘none’ vs the ‘moderate/advanced (T4a + b)’ class and ‘minimal (T3)’ vs ‘moderate/advanced (T4a + b)’ (*P* = 0.018 and *P* = 0.039, respectively Fig. 2E). We also observed a statistical significant decrease (from a median of 3.8 to 0.8; *P* = 0.002) of the *SLC5A5* expression in cases with new tumor events (Fig. 2F), lumping together 12 cases of distant metastasis (6 lung; 1 lung + bone; 1 lymph node only; 1 lung + femur + neck + pleura + liver; 1 bone; 2 unknown) and 14 locoregional recurrences (10 lymph node only; 2 left thyroid; 1 lymph node + soft tissue; 1 unknown). Finally, *SLC5A5* expression was significantly higher in the absence (median = 21.77) of the evaluated mutations: *RAS* (*P* = 0.034), *TERT*p (*P* = 0.0072) and *BRAF*V600E (*P* = 3.1 × 10^−8^). The PTCs that harbored only *TERT*p, only *BRAF* or simultaneous *TERT*p and *BRAF* mutations displayed significantly lower expression of *SLC5A5* than the WT tumors. The group with *RAS* mutations displayed the second highest expression value (median = 7.50), reaching statistical significance when compared with the groups including *BRAF* mutation only and *BRAF* + *TERT*p mutations (median = 2.27 in *BRAF* (*P* = 0.042); median = 1.89 in *TERT*+*BRAF* (*P* = 0.027)) (Fig. 2G).

### NIS expression

**Figure 3:**
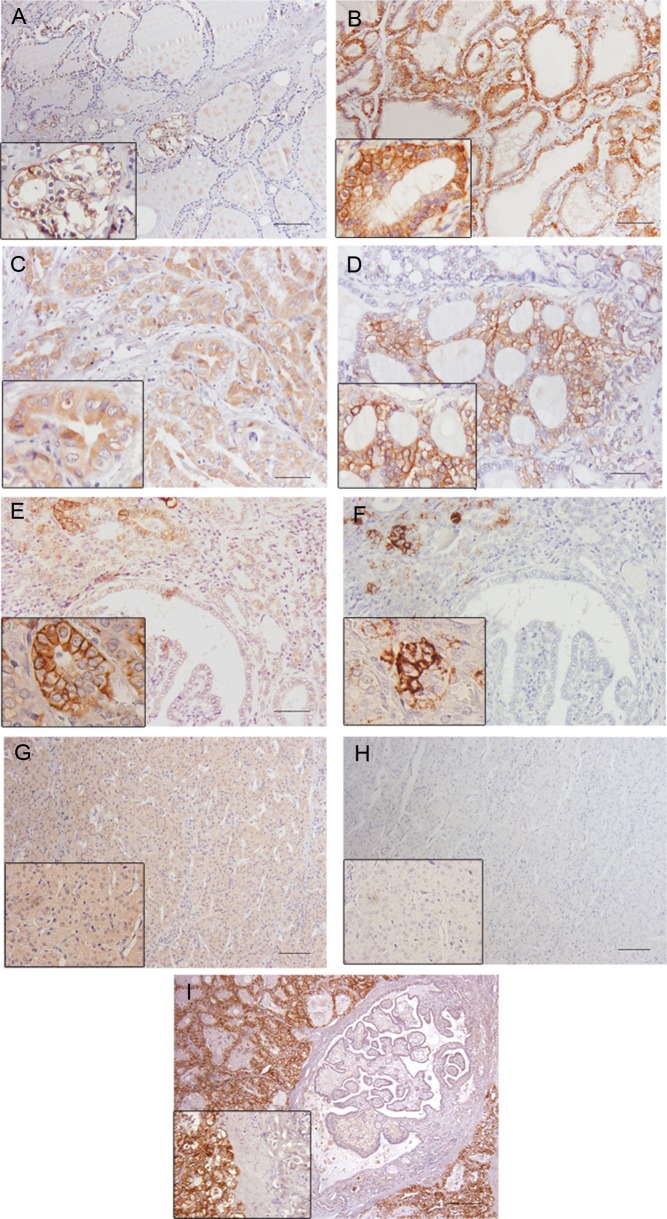
NIS immunoexpression in different thyroid tissues. (A) Normal thyroid; (B) Graves’ disease; (C) cytoplasmatic staining in an oncocytic PTC; (D) membrane staining in a fvPTC; (E and F) NIS immunoexpression in a fvPTC without and with TSA amplification signal, respectively; (G and H) NIS immunoexpression in a FTC without and with TSA amplification signal, respectively; (I) negative staining in a cPTC and strong membrane staining in the surrounding Graves’ disease. In F and H, notice the loss of cytoplasmatic staining after the use of TSA amplification system. Bar 100 μm.

In normal thyroid tissues, NIS immunohistochemical expression was mainly localized in the basolateral plasma membrane of follicular cells. NIS positivity was detected in a few foci of isolated follicles throughout the tissue and within the positive follicles most of the cells were positive. Positivity was more frequently detected in small follicles composed by cuboidal and columnar cells and rarely detected in large follicles limited by flattened cells (Fig. 3A). In Graves’ disease, NIS was widely expressed and present in the basolateral plasma membrane of the great majority of follicular cells (Fig. 3B). In carcinomas, NIS staining was observed in 71.6% of the cases (74.8% of cPTCs, 69.8% of fvPTCs, 80.9% of other PTC variants, 55% of FTC and 67% of PDTC). Its location was predominantly in the cytoplasm (124/211) (Fig. 3C) and nucleus (15/211) and only 12/211 of the cases presented NIS in the basolateral plasma membrane of tumor cells (Fig. 3D).

Since we observed a low percentage of carcinomas with NIS staining in the basolateral membrane, we hypothesized that our IHC approach was not being sensitive enough to detect small amounts of NIS. To clarify this issue, we used two strategies: a TSA signal amplification method and the use of another NIS antibody characterized by a different specificity compared to the commercial antibody (36).

The TSA signal amplification method was applied in a subset of 44 carcinomas with different staining patterns (16 with cytoplasmic staining in the tumor and membrane staining in adjacent thyroid; 3 with membrane staining in the tumor; 5 negative both in the in tumor and the adjacent thyroid and, finally, 20 with only cytoplasmic staining in tumor and adjacent thyroid). When we compared the slides performed with and without TSA signal amplification, we verified that only the membrane staining remained and appeared more intense with the amplification method. In these cases, the staining involved almost always the same foci of cells that already presented membrane staining (Fig. 3E, F, G and H) i.e. it did not stain additional cells. The intra-cytoplasmic staining vanished both in cancer and in normal tissues. Furthermore, we performed IHC using a homemade antibody for human NIS, pAb 795 against a peptide corresponding to the C-terminal sequence of hNIS pAb 795 (36) in 24 carcinomas (12 cPTC, 4 fvPTC, 2 micro PTC, 2 tall cell PTC, 2 FTC and 2 PDTC). The results were similar to those obtained with clone FP5A (Thermo Scientific/Lab Vision).

Since some doubts remained about the specificity of the cytoplasmic staining, and because NIS is only active when present in the basolateral membrane of the cells, we performed statistical analysis dividing our series in two groups: with and without membrane staining.

We did not find any significant association between NIS expression in the membrane and age, tumor size, tumor capsule, multifocality, lymphocytic infiltration, vascular invasion, lymph node metastases, tumor margins, distant metastases, staging, *BRAF*, *NRAS* and *TERT*p status, additional treatments, disease-free status at one year, disease-free status at the end of follow-up or disease-specific survival in the DTC group. When we analyzed NIS expression between WT PTCs and those harboring any of the studied mutations, we verified that NIS-positive expression was significantly more frequent in WT PTCs (*P* = 0.01) (Table 2). The number of RAI therapies, as well as the cumulative dose of RAI, did not differ significantly between patients with or without NIS expression in the basolateral membrane of primary tumor’s cells.

**Table 2:** Associations between NIS expression and clinicopathological and molecular features in PTCs.

	**NIS immunoexpression**		***P* value**
	Negative	Positive	
Genetic background *n* = 118			
WT	45 (41.3%)	8 (88.9%)	
Mutated^#^	64 (58.7%)	1 (11.1%)	0.011

^#^*BRAF, NRAS* or *TERT*p mutations.

The thorough analysis of the few cases with membrane staining (*n* = 12) revealed that all but one carcinoma were wild type for the studied mutations (*NRAS*, *BRAF* or *TERT*p). These cases presented variable outcomes i.e. presence of distant metastases, number of RAI therapies, cumulative dose of RAI, the need of additional treatments, disease-free status and death (disease caused), that are apparently unrelated with the presence of NIS membrane expression (Table 3).

**Table 3:** Clinicopathological and molecular data of cases presenting NIS membrane staining.

	**Diagnosis**	***BRAF***	***NRAS***	***TERT*p**	**Lymph node metastases**	**Distant metastases**	**Number of ^131^I therapies**	**Cumulative dose** (mCi)	**Aditional treatments**	One year DFS*	DFS*^,#^	**Deaths**
Case 1	cPTC	WT	WT	WT	No	Bone	3	457.5	No	No	No	No
Case 2	cPTC	WT	WT	WT	No	No	1	63	No	Yes	Yes	No
Case 3	cPTC	WT	WT	WT	Yes	No	3	459	2 surgeries	No	No	No
Case 4	fvPTC	WT	WT	WT	Yes	No	1	37	No	Yes	Yes	No
Case 5	fvPTC	WT	WT	WT	No	No	2	382	No	No	No	No
Case 6	PDTC	WT	WT	WT	No	Lung + bone	5	798	2 surgeries	Yes	No	No
Case 7	cPTC	WT	WT	124G>A	Yes	No	4	527	U/I	No	No	No
Case 8	sclPTC	WT	WT	WT	Yes	No	3	400	U/I	No	No	No
Case 9	FTC	WT	WT	WT	No	No	1	102	U/I	U/I	Yes	No
Case 10	cPTC	WT	WT	WT	Yes	U/I	U/I	U/I	U/I	U/I	U/I	U/I
Case 11	fvPTC	WT	WT	WT	U/I	U/I	U/I	U/I	U/I	U/I	U/I	U/I
Case 12	cPTC	WT	WT	WT	Yes	U/I	U/I	U/I	U/I	U/I	U/I	U/I

*DFS disease-free survival; ^#^at the end of follow-up.

sclPTC, sclerosing variant of PTC; U/I, unavailable information.

## Discussion

In this work, we tried to clarify the impact of NIS expression (mRNA and protein) on thyroid tumors’ aggressiveness and therapy success and, as a result of the above, the putative prognostic significance of *SLC5A5* mRNA and NIS protein expression. Moreover, we also addressed the impact of the genetic background of the tumor on *SLC5A5* and NIS expression as well as its targeting to the basolateral cell membrane.

We found that *SLC5A5* expression was always lower in tumors than in normal adjacent counterparts as reported by other groups (6, 7, 37). We observed a significantly lower *SLC5A5* expression in male gender patients, and in cases with vascular invasion, as well as a tendency to lower *SLC5A5* expression in cases with extrathyroidal extension, but no differences were found in cases with and without lymph node metastases (Table 1). When we compare the results from our series to those from TCGA data, we confirmed that tumors express significantly less *SLC5A5* compared to normal adjacent tissue, that *SLC5A5* was not differently expressed in the presence or absence of lymph node metastases (at the time of diagnosis) and a significant lower *SLC5A5* expression was found in tumors with extrathyroidal extension (moderate/advanced) compared to those without extrathyroidal extension (Fig. 2D). However, the differential expression of *SLC5A5* between genders was not confirmed (Fig. 2B). Unfortunately, in TCGA database, there was no information about vascular invasion, so we could not validate this result in this large series.

The significantly lower *SLC5A5* expression in cases presenting vascular invasion and extrathyroidal extension suggests that a decreased *SLC5A5* expression may be associated to an aggressive tumor behavior and thus may help to characterize patients at risk for poor therapy response. Further analysis of TCGA data demonstrated that *SLC5A5* expression is significantly lower in cases that had locoregional recurrences and/or distant metastases (Fig. 2E). Given the high prognostic impact of recurrences and distant metastases (38), these results suggest that a lower expression of *SLC5A5* in thyroid primary tumor seems to be associated with features of higher aggressiveness of the primary tumor and also with a worse prognosis and with poor response to therapy. Two groups reported that *SLC5A5* was significantly less expressed in DTCs larger than 2 cm and PTCs larger than 1 cm (in comparison to ≤2 cm and <1 cm, respectively) (1, 39), TCGA results corroborated the literature by showing that larger PTCs (>2 cm) expressed significantly less *SLC5A5* compared to those with ≤2 cm. Since larger tumors are associated with higher recurrence rates and worse prognosis (40, 41), the significantly lower *SLC5A5* expression in tumors larger than 2 cm may be considered as an additional fact linking lower *SLC5A5* mRNA expression with higher tumor aggressiveness. Nevertheless, we must interpret this information carefully: larger tumors may present higher levels of necrosis/fibrosis and also additional non-carcinomatous tissue as part of the nodule, which may contribute to a lower *SLC5A*5 expression. In our series, we did not include microcarcinomas, so the group of tumors with ≤2 cm was very small, precluding any meaningful analysis (data not shown).

Previous studies reported a lower *SLC5A5* expression in cases harboring *BRAF*V600E, and there is experimental evidence showing that *BRAF*V600E can impair *SLC5A5* expression (1, 19, 27, 39), nevertheless the impact of other relevant mutations found in thyroid tumors on *SLC5A5* expression remained unknown. In our series, *SLC5A5* expression was lower but did not reach statistical significance in the *BRAF*V600E PTC compared to that of *BRAF* wild-type group. The lack of significance in our series may be due to differences in size and composition of the series, since the above mentioned studies addressing *SLC5A5* expression and *BRAF* V600E (1, 19, 27, 39) used larger series of PTC.

When we compared *SLC5A5* expression (retrieved from TCGA database) between PTCs harboring different mutations (*BRAF*V600E, *TERT*p and *RAS*) and WT PTCs, we observed that independently of the mutation, *SLC5A5* expression was always significantly lower compared to WT PTCs. Moreover, we also observed that *RAS* mutation was the one with lower impact on *SLC5A5* expression. PTCs with *RAS* mutation displayed significantly higher levels of *SLC5A5* compared to *BRAF*V600E and *BRAF* + *TERT*p-mutated PTCs. In fact, it has been previously reported that a distinct profile of expression of genes involved in thyroid hormone biosynthesis (being *SLC5A5* one of these genes) between *BRAF*V600E and *RAS*-driven PTCs, with *RAS*-like PTCs having relatively high thyroid differentiation score (30).

Our results on the immunohistochemical NIS expression in normal thyroid and Graves’ disease (an autoimmune condition known to express high levels of NIS) (42) were in accordance to data previously reported (10, 12, 14, 17, 21), i.e., focal membrane expression of NIS in normal thyroid gland and a strong and widespread membrane NIS expression in Graves’ disease. The great difference observed in NIS expression between normal thyroid and in Graves’ disease may be considered as an example of how TSH is able to regulate NIS expression and the targeting to the membrane. Regarding carcinomas, we observed that the majority (71.6%) displayed NIS immunostaining, which is in accordance to the literature (13, 14, 17, 18, 19, 20, 21, 22, 23) (Table 4), but only a minority presented NIS in the basolateral membrane of tumor cells (5.7%). If one compares the percentage of cases with NIS plasma membrane staining, there are large differences between studies (Table 4). Such differences may be due to the variable size of the series and to differences in the antibodies used to perform the IHC (almost every study uses its own antibody, Table 4). In fact, one study compared NIS immunostaining using two different antibodies in a large series of thyroid carcinomas and observed some differences in the percentage of positive cells (29). To be sure that we were not missing any signal, we performed the IHC for NIS with TSA signal amplification in a subset of carcinomas with different immunostaining patterns and observed a complete vanish of intracytoplasmic staining and an amplification of the membrane staining. These results, like those from Peyrottes and coworkers (21) rise some questions about the real significance of NIS intracytoplasmic staining, so we decided to perform our analysis considering positive only the cases with membrane staining.

**Table 4:** Bibliographic revision and present results of NIS protein evaluation by IHC in thyroid carcinomas.

**Reference**	**No. of carcinomas**	**Anti-NIS antibodies used in the study**	**Negative cases** (%)	**Positive cases** (%)*	**Cases with membrane staining** (%)**
(26)	4 DTCs	Produced by authors			
(10)	14 DTCs	Produced by authors	Descriptive studies		
(11)	12 PTCs	Produced by authors			
(12)	9 DTCs	Produced by authors			
(13)	60 DTCs	Clone FP-13	26.7	73.3	N/A
(14)	57 (53 DTCs; 2 ATC; 2 MTC)	Produced by authors	29.8	70.2	15.8
(15)	67 DTCs	Donated by Dr SM Jhiang of Ohio State University, USA	67.2	32.8	N/A
(17)	90 (87 DTCs; 3 ATC)	Produced by authors	22.5	77.5	Some
(24)	67 PTCs	Pohlenz *et al*. (2000)	N/A		Some
(18)	17 PTCs	Clone Ab-1	0	100	58.8
(19)	40 PTCs	Brahms Diagnostica GmbH, Berlin, Germany	0	100	52.5
(20)	29 (25 DTCs; 4 ATC)	Clone FP5A	37.5	62.5	N/A
(21)	47 (42 DTCs; 5MTC)	Clones 39S, Ab-1 and FP5A	49	51	0
(25)	50 PTCs	Tazebay *et al*. (2000)	N/A		8
(22)	32 DTCs	Zhongshan Goldbridge Biotechnology, Beijin China	0	100	18.8
(1)^#^	265 DTCs	Clone FP5A	88	12	12
(29)^#^	380 (283 DTCs; 97 ATCs)	Donated by N. Morgenthaler (Brahms Diagnostics, Henningsdorf, Germany)	87.4	12.6	12.6
(23)	370 PTCs	Clone SPM186	32.7	67.3	0.8
(present study)	211 (199 DTCs; 12 PDTCs)	Clone FP5A	28.4	71.6	5.7

*Percentage of positive cases (independently of the cellular location); **percentage of cases with NIS membrane staining with or without simultaneous cytoplasmic staining; ^#^these specific studies only considered positive cases with membrane staining.

ATC, anaplastic thyroid carcinoma; MTC, medullary thyroid carcinoma; N/A, not addressed; PDTC, poorly differentiated thyroid carcinoma.

The presence of NIS in the basolateral membrane of thyroid primary carcinomas did not associate with clinicopathological features, response to therapy or prognosis. If we look to the treatment of thyroid carcinoma (surgery followed by RAI ablation), only the remnants, metastases and eventually the recurrences are subjected to RAI. Prior to RAI ablation patients are subjected to TSH stimulation, either by withdrawal of thyroid hormones or by the administration of recombinant TSH (2). Since TSH has a major role in NIS expression and targeting to the membrane (43), we can hypothesize that levels of membrane NIS in stimulated recurrences and metastases may be different from those in non-stimulated primary tumors because they may reflect two different biological conditions. It seems pertinent that further studies of NIS expression should be performed in recurrences and/or remaining thyroid tissues rather than in the primary tumors. In fact, one study addressed NIS expression in nontumoral thyroid tissue and reported a significant association between lower NIS expression and delayed structural response (3). Even though the nontumoral tissue used in this study was obtained at the time of surgery (normal TSH levels), this might corroborate our hypothesis. Altogether, this information may help to explain why NIS expression in the primary tumor does not predict RAI therapy success and/or prognosis.

Another interesting finding of our study was the observation that the cases with NIS membrane staining were predominantly wild type for the analyzed mutations (*NRAS*, *BRAF* and *TERT*p) (Table 3). Although this membrane expression was not associated with any outcome (clinicopathological features or prognosis), it is tempting to advance that the genetic background of tumors influence NIS targeting to the membrane. There are *in vitro* evidence that *BRAF*V600E mutation affects NIS targeting to the membrane (24), but the impact of the other mutations (*NRAS* and *TERT*p) remains unknown.

In summary, the absence of *BRAF* and *NRAS* mutations in every carcinoma displaying NIS membrane staining at immunohistochemistry supports the assumption that the genetic background of tumors may be of major importance to *SLC5A5* expression as well as to NIS targeting to the basolateral membrane. On the other hand, NIS immunohistochemical expression did not predict tumor behavior, therapy response or outcome. Moreover, *SLC5A5* mRNA expression was significantly lower in mutated PTCs and a lower *SLC5A5* mRNA expression was associated with tumor aggressiveness and worse prognosis. Thus, the study of *SLC5A5* mRNA expression is much more informative compared to NIS expression evaluated by IHC.

## Supplementary Material

Supporting Table 1

## Declaration of interest

The authors declare that there is no conflict of interest that could be perceived as prejudicing the impartiality of the research reported.

## Funding

This study was supported by FCT (‘Portuguese Foundation for Science and Technology’) through PhD grants to Catarina Tavares (SFRH/BD/87887/2012), Ana Pestana (SFRH/BD/110617/2015), Rui Batista (SFRH/BD/111321/2015) and by a CNPq PhD grant (‘National Counsel of Technological and Scientific Development’, Brazil), Science without Borders, Process n# 237322/2012-9 for Luciana Ferreira. Miguel Melo received a grant from Genzyme for the research project ‘Molecular biomarkers of prognosis and response to therapy in differentiated thyroid carcinomas’. Further funding was obtained from FEDER – Fundo Europeu de Desenvolvimento Regional funds through the COMPETE 2020 – Operational Program for Competitiveness and Internationalization (POCI), Portugal 2020, and by Portuguese funds through FCT – Fundação para a Ciência e a Tecnologia/Ministério da Ciência, Tecnologia e Inovação in the framework of the project ‘Institute for Research and Innovation in Health Sciences’ (POCI-01-0145-FEDER-007274) and by the project ‘Advancing cancer research: from basic knowledgement to application’; NORTE-01-0145-FEDER-000029; ‘Projetos Estruturados de I&D&I’, funded by Norte 2020-Programa Operacional Regional do Norte. This work was also financed by Sociedade Portuguesa de Endocrinologia Diabetes e Metabolismo through a grant ‘Prof. E Limbert Sociedade Portuguesa de Endocrinologia Diabetes e Metabolismo/Sanofi-Genzyme in thyroid pathology’.

## References

[1] Morari EC Marcello MA Guilhen AC Cunha LL Latuff P Soares FA Vassallo J Ward LS. Use of sodium iodide symporter expression in differentiated thyroid carcinomas. Clinical Endocrinology 2011 75 247–254. (10.1111/j.1365-2265.2011.04032.x) 21521301

[2] Haugen BR Alexander EK Bible KC Doherty GM Mandel SJ Nikiforov YE Pacini F Randolph GW Sawka AM Schlumberger M 2015 American Thyroid Association Management Guidelines for adult patients with thyroid nodules and differentiated thyroid cancer: the American Thyroid Association Guidelines Task Force on Thyroid Nodules and Differentiated Thyroid Cancer. Thyroid 2016 26 1–133. (10.1089/thy.2015.0020) PMC4739132 26462967

[3] Yildirim-Poyraz N Yazgan A Ozdemir E Gozalan A Keskin M Ersoy R Turkolmez S Cakir B. Predictive role of nontumoral sodium iodide symporter activity and preoperative thyroid characteristics in remission process of thyroid cancer patients. Annals of Nuclear Medicine 2014 28 623–631. (10.1007/s12149-014-0854-5) 24823701

[4] Arturi F Russo D Schlumberger M du Villard JA Caillou B Vigneri P Wicker R Chiefari E Suarez HG Filetti S. Iodide symporter gene expression in human thyroid tumors. Journal of Clinical Endocrinology and Metabolism 1998 83 2493–2496. (10.1210/jcem.83.7.4974) 9661633

[5] Lazar V Bidart JM Caillou B Mahé C Lacroix L Filetti S Schlumberger M. Expression of the Na+/I- symporter gene in human thyroid tumors: a comparison study with other thyroid-specific genes. Journal of Clinical Endocrinology and Metabolism 1999 84 3228–3234. (10.1210/jcem.84.9.5996) 10487692

[6] Arturi F Russo D Bidart JM Scarpelli D Schlumberger M Filetti S. Expression pattern of the pendrin and sodium/iodide symporter genes in human thyroid carcinoma cell lines and human thyroid tumors. European Journal of Endocrinology 2001 145 129–135. (10.1530/eje.0.1450129) 11454507

[7] Mian C Barollo S Pennelli G Pavan N Rugge M Pelizzo MR Mazzarotto R Casara D Nacamulli D Mantero F Molecular characteristics in papillary thyroid cancers (PTCs) with no 131I uptake. Clinical Endocrinology 2008 68 108–116. (10.1111/j.1365-2265.2007.03008.x) 17854396

[8] Arturi F Russo D Giuffrida D Schlumberger M Filetti S. Sodium-iodide symporter (NIS) gene expression in lymph-node metastases of papillary thyroid carcinomas. European Journal of Endocrinology 2000 143 623–627. (10.1530/eje.0.1430623) 11078986

[9] Brose MS Smit J Capdevila J Elisei R Nutting C Pitoia F Robinson B Schlumberger M Shong YK Takami H. Regional approaches to the management of patients with advanced, radioactive iodine-refractory differentiated thyroid carcinoma. Expert Review of Anticancer Therapy 2012 12 1137–1147. (10.1586/era.12.96) 23098114

[10] Caillou B Troalen F Baudin E Talbot M Filetti S Schlumberger M Bidart JM. Na+/I- symporter distribution in human thyroid tissues: an immunohistochemical study. Journal of Clinical Endocrinology and Metabolism 1998 83 4102–4106. (10.1210/jcem.83.11.5262) 9814499

[11] Saito T Endo T Kawaguchi A Ikeda M Katoh R Kawaoi A Muramatsu A Onaya T. Increased expression of the sodium/iodide symporter in papillary thyroid carcinomas. Journal of Clinical Investigation 1998 101 1296–1300. (10.1172/JCI1259) 9525971 PMC508706

[12] Castro MR Bergert ER Beito TG McIver B Goellner JR Morris JC. Development of monoclonal antibodies against the human sodium iodide symporter: immunohistochemical characterization of this protein in thyroid cells. Journal of Clinical Endocrinology and Metabolism 1999 84 2957–2962. (10.1210/jcem.84.8.5871) 10443704

[13] Castro MR Bergert ER Goellner JR Hay ID Morris JC. Immunohistochemical analysis of sodium iodide symporter expression in metastatic differentiated thyroid cancer: correlation with radioiodine uptake. Journal of Clinical Endocrinology and Metabolism 2001 86 5627–5632. (10.1210/jcem.86.11.8048) 11701745

[14] Dohan O Baloch Z Banrevi Z Livolsi V Carrasco N. Rapid communication: predominant intracellular overexpression of the Na(+)/I(-) symporter (NIS) in a large sampling of thyroid cancer cases. Journal of Clinical Endocrinology and Metabolism 2001 86 2697–2700. (10.1210/jcem.86.6.7746) 11397873

[15] Min JJ Chung JK Lee YJ Jeong JM Lee DS Jang JJ Lee MC Cho BY. Relationship between expression of the sodium/iodide symporter and 131I uptake in recurrent lesions of differentiated thyroid carcinoma. European Journal of Nuclear Medicine and Molecular Imaging 2001 28 639–645. (10.1007/s002590100509) 11383871

[16] Tonacchera M Viacava P Agretti P de Marco G Perri A di Cosmo C de Servi M Miccoli P Lippi F Naccarato AG Benign nonfunctioning thyroid adenomas are characterized by a defective targeting to cell membrane or a reduced expression of the sodium iodide symporter protein. Journal of Clinical Endocrinology and Metabolism 2002 87 352–357. (10.1210/jcem.87.1.8173) 11788674

[17] Wapnir IL van de Rijn M Nowels K Amenta PS Walton K Montgomery K Greco RS Dohán O Carrasco N. Immunohistochemical profile of the sodium/iodide symporter in thyroid, breast, and other carcinomas using high density tissue microarrays and conventional sections. Journal of Clinical Endocrinology and Metabolism 2003 88 1880–1888. (10.1210/jc.2002-021544) 12679487

[18] Lee SJ Choi KC Han JP Park YE Choi MG. Relationship of sodium/iodide symporter expression with I131 whole body scan uptake between primary and metastatic lymph node papillary thyroid carcinomas. Journal of Endocrinological Investigation 2007 30 28–34. (10.1007/BF03347392) 17318019

[19] Romei C Ciampi R Faviana P Agate L Molinaro E Bottici V Basolo F Miccoli P Pacini F Pinchera A BRAFV600E mutation, but not RET/PTC rearrangements, is correlated with a lower expression of both thyroperoxidase and sodium iodide symporter genes in papillary thyroid cancer. Endocrine-Related Cancer 2008 15 511–520. (10.1677/ERC-07-0130) 18509003

[20] Jung YH Hah JH Sung MW Kim KH Cho SY Jeon YK. Reciprocal immunohistochemical expression of sodium/iodide symporter and hexokinase I in primary thyroid tumors with synchronous cervical metastasis. Laryngoscope 2009 119 541–548. (10.1002/lary.20073) 19235753

[21] Peyrottes I Navarro V Ondo-Mendez A Marcellin D Bellanger L Marsault R Lindenthal S Ettore F Darcourt J Pourcher T. Immunoanalysis indicates that the sodium iodide symporter is not overexpressed in intracellular compartments in thyroid and breast cancers. European Journal of Endocrinology 2009 160 215–225. (10.1530/EJE-08-0505) 19029227

[22] Wang ZF Liu QJ Liao SQ Yang R Ge T He X Tian CP Liu W. Expression and correlation of sodium/iodide symporter and thyroid stimulating hormone receptor in human thyroid carcinoma. Tumori 2011 97 540–546. (10.1700/950.10410) 21989446

[23] Wei S Gao M Zhao C Pan Y Li H Li J Li X. Low expression of sodium iodide symporter expression in aggressive variants of papillary thyroid carcinoma. International Journal of Clinical Oncology 2014 19 800–804. (10.1007/s10147-013-0620-z) 24096868

[24] Riesco-Eizaguirre G Gutierrez-Martinez P Garcia-Cabezas MA Nistal M Santisteban P. The oncogene BRAF V600E is associated with a high risk of recurrence and less differentiated papillary thyroid carcinoma due to the impairment of Na+/I- targeting to the membrane. Endocrine-Related Cancer 2006 13 257–269. (10.1677/erc.1.01119) 16601293

[25] Riesco-Eizaguirre G Rodriguez I De la Vieja A Costamagna E Carrasco N Nistal M Santisteban P. The BRAFV600E oncogene induces transforming growth factor beta secretion leading to sodium iodide symporter repression and increased malignancy in thyroid cancer. Cancer Research 2009 69 8317–8325. (10.1158/0008-5472.CAN-09-1248) 19861538

[26] Jhiang SM Cho JY Ryu KY DeYoung BR Smanik PA McGaughy VR Fischer AH Mazzaferri EL. An immunohistochemical study of Na+/I- symporter in human thyroid tissues and salivary gland tissues. Endocrinology 1998 139 4416–4419. (10.1210/endo.139.10.6329) 9751526

[27] Durante C Puxeddu E Ferretti E Morisi R Moretti S Bruno R Barbi F Avenia N Scipioni A Verrienti A BRAF mutations in papillary thyroid carcinomas inhibit genes involved in iodine metabolism. Journal of Clinical Endocrinology and Metabolism 2007 92 2840–2843. (10.1210/jc.2006-2707) 17488796

[28] Yazgan A Yildirim N Gozalan A Gumustas S Kilicarslan A Balci S Aydin C Ersoy R Cakir B Guler G. The correlation of sodium iodide symporter and BRAF(V600E) mutation in classical variant papillary thyroid carcinoma. Annals of Diagnostic Pathology 2016 22 58–62. (10.1016/j.anndiagpath.2016.04.002) 27180062

[29] Kollecker I von Wasielewski R Langner C Muller JA Spitzweg C Kreipe H Brabant G. Subcellular distribution of the sodium iodide symporter in benign and malignant thyroid tissues. Thyroid 2012 22 529–535. (10.1089/thy.2011.0311) 22545753

[30] Cancer Genome Atlas Research Network. Integrated genomic characterization of papillary thyroid carcinoma. Cell 2014 159 676–690. (10.1016/j.cell.2014.09.050) 25417114 PMC4243044

[31] DeLellis RA Lloyd RV Heitz PU Eng C. WHO Classification of Tumours. Pathology and Genetics of Tumours of Endocrine Organs. Lyon, France: IARC Press, 2004.

[32] Trovisco V Soares P Preto A de Castro IV Lima J Castro P Maximo V Botelho T Moreira S Meireles AM Type and prevalence of BRAF mutations are closely associated with papillary thyroid carcinoma histotype and patients' age but not with tumour aggressiveness. Virchows Archivs 2005 446 589–595. (10.1007/s00428-005-1236-0) 15902486

[33] Melo M da Rocha AG Vinagre J Batista R Peixoto J Tavares C Celestino R Almeida A Salgado C Eloy C TERT promoter mutations are a major indicator of poor outcome in differentiated thyroid carcinomas. Journal of Clinical Endocrinology and Metabolism 2014 99 E754–E765. (10.1210/jc.2013-3734) 24476079 PMC4191548

[34] Vinagre J Almeida A Populo H Batista R Lyra J Pinto V Coelho R Celestino R Prazeres H Lima L Frequency of TERT promoter mutations in human cancers. Nature Communications 2013 4 2185 (10.1038/ncomms3185) 23887589

[35] Tavares C Coelho MJ Melo M da Rocha AG Pestana A Batista R Salgado C Eloy C Ferreira L Rios E pmTOR is a marker of aggressiveness in papillary thyroid carcinomas. Surgery 2016 160 1582–1590. (10.1016/j.surg.2016.06.050) 27574774

[36] Trouttet-Masson S Selmi-Ruby S Bernier-Valentin F Porra V Berger-Dutrieux N Decaussin M Peix JL Perrin A Bournaud C Orgiazzi J Evidence for transcriptional and posttranscriptional alterations of the sodium/iodide symporter expression in hypofunctioning benign and malignant thyroid tumors. American Journal of Pathology 2004 165 25–34. (10.1016/S0002-9440(10)63272-5) 15215159 PMC1618532

[37] Park HJ Park KY Gong G Hong SJ Ahn IM. Expressions of human sodium iodide symporter mRNA in primary and metastatic papillary thyroid carcinomas. Thyroid 2000 10 211–217. (10.1089/thy.2000.10.211) 10779135

[38] Soares P Celestino R Melo M Fonseca E Sobrinho-Simoes M. Prognostic biomarkers in thyroid cancer. Virchows Archivs 2014 464 333–346. (10.1007/s00428-013-1521-2) 24487783

[39] Bastos AU Oler G Nozima BH Moyses RA Cerutti JM. BRAF V600E and decreased NIS and TPO expression are associated with aggressiveness of a subgroup of papillary thyroid microcarcinoma. European Journal of Endocrinology 2015 173 525–540. (10.1530/EJE-15-0254) 26338373

[40] Jukkola A Bloigu R Ebeling T Salmela P Blanco G. Prognostic factors in differentiated thyroid carcinomas and their implications for current staging classifications. Endocrine-Related Cancer 2004 11 571–579. (10.1677/erc.1.00826) 15369456

[41] Shah JP Loree TR Dharker D Strong EW Begg C Vlamis V. Prognostic factors in differentiated carcinoma of the thyroid gland. American Journal of Surgery 1992 164 658–661. (10.1016/S0002-9610(05)80729-9) 1463119

[42] Bartalena L Chiovato L Vitti P. Management of hyperthyroidism due to Graves' disease: frequently asked questions and answers (if any). Journal of Endocrinological Investigation 2016 39 1105–1114. (10.1007/s40618-016-0505-x) 27319009

[43] Riesco-Eizaguirre G Santisteban P. A perspective view of sodium iodide symporter research and its clinical implications. European Journal of Endocrinology 2006 155 495–512. (10.1530/eje.1.02257) 16990649

