# Supplementary material for: NIS expression in thyroid tumors, relation with prognosis clinicopathological and molecular features
Source: Endocr Connect. 2017 Nov 9;7(1):78–90. doi: 10.1530/EC-17-0302 (PMC5754505; doi:10.1530/EC-17-0302)
Supplement: Supporting Table 1 [file ec-7-78-t001.pdf]

Supplementary data

Supplementary Table 1.Clinicopathological data of the 229 patients with carcinomas included in the study.

	Total and (%)	PTC	FTC	PDTC
**Age (n)**	226	191	22	13
≥45 years	115(50.9)	91(47.6)	13(59.0)	11(84.6)
**Gender (n)**	228	193	22	13
Male	50(21.9)	39(20.2)	6(27.3)	5(38.5)
**Tumor size (n)**	212	181	20	11
≥2cm	161(75.9)	130(71.8)	20(100)	11(100)
**Tumor capsule (n)**	203	175	18	10
Present	108(53.2)	84(48.0)	18(100)	6(60)
**Tumor capsule invasion (n)**	109	86	17	6
Yes	90(82.6)	67(77.9)	17(100)	6(100)
**Extrathyroidal extension (n)**	196	170	18	8
Present	78(39.8)	76(44.7)	0(0)	2(25)
**Multifocality (n)**	198	174	15	9
Multifocal	86(43.4)	73(42.0)	9(60.0)	4(44.4)
**Lymphocytic infiltrate (n)**	197	170	18	9
Present	111(56.3)	102(60.0)	6(33.3)	3(33.3)
**Lymph node metastases (n)**	186	160	16	10
Present	93(50)	89(55.6)	0(0)	4(40.0)
**Vascular invasion (n)**	198	172	18	8
Present	80(40.4)	64(37.2)	9(50.0)	7(87.5)
**Tumor margins (n)**	124	114	5	5
Infiltrative	83(66.9)	81(71.1)	1(20.0)	1(20.0)
**Distant metastases (n)**	145	128	9	8
Present	33(22.8)	22(17.2)	5(55.6)	6(75.0)
**Staging (AJCC) (n)**	118	107	5	6
I	68(57.6)	65(60.8)	1(20.0)	2(33.3)
II	10(8.5)	7(6.5)	2(40.0)	1(16.7)
III	29(24.6)	26(24.3)	1(20.0)	2(33.3)
IV	11(9.3)	9(8.4)	1(20.0)	1(16.7)
**One year disease free (n)**	136	122	7	7
No	64(47.1)	56(45.9)	6(85.7)	2(28.6)
**Disease-free (end of follow up) (n)**	141	125	8	8
No	61(43.3)	51(40.8)	6(75.0)	4(50.0)
**Deaths (n)**	146	129	9	8
Yes	9(6.2)	5(3.9)	1(11.1)	3(37.5)
***BRAF* (n)**	226	191	22	13
V600E	82(36.3)	79(41.4)	0(0)	3(23.1)
***NRAS* (n)**	207	176	19	12
Mutation	12(5.8)	8(4.5)	3(15.8)	1(8.3)
***TERT*p mutation (n)**	201	168	20	13
Mutation	19(9.5)	11(6.5)	5(25.0)	3(23.1)
